# Response to “Three drugs are unnecessary for treating paucibacillary leprosy—A critique of the WHO guidelines”

**DOI:** 10.1371/journal.pntd.0008169

**Published:** 2020-06-04

**Authors:** Bhushan Kumar, Vishal Thakur, Tarun Narang, Sunil Dogra

**Affiliations:** 1 Consultant Dermatologist Shalby Hospital, SAS Nagar, Punjab; 2 Department of Dermatology, Venereology, and Leprology; Postgraduate Institute of Medical Education and Research, Chandigarh, India; Swiss Tropical and Public Health Institute, SWITZERLAND

We read with interest the viewpoint by Lockwood and colleagues on the recent World Health Organization (WHO) guidelines recommending three drugs for treating paucibacillary (PB) leprosy. [1] Out of the various other issues raised by the authors (namely, diagnostic tests, reactions, stigma, and disability), we would like to submit our perception only on the issue of addition of clofazimine to this recommended therapeutic regimen. [2]

Effectiveness of a drug regimen in any infectious disease is based on two important factors: incidence of relapse and amelioration of sign and symptoms. Authors refer to the already low figures of relapse for PB leprosy as reported by WHO (1.07% for PB and 0.77% for multibacillary [MB] leprosy) after 9 years of the release from treatment (RFT); interestingly, the relapse rate is higher in PB leprosy. Indian studies have also reported a higher relapse rate for PB leprosy in comparison to MB disease but higher than what is projected by WHO. Grugni et al. found relapse rate of 5.63% (17.5 per 1000 person years at risk) in PB leprosy. [3] Various other studies using person years of observation have also noted relapse rates varying from 0.65% to 3.0% for PB and 0.02% to0.8% for MB leprosy. [4,5] An earlier study with 16-year follow-up observed a crude cumulative relapse rate of 1.78% and relapse rates of PB and MB as 1.9% and 0.84%, respectively. [6] So, it can be safely presumed that the occurrence of higher relapse rate in PB leprosy in comparison to MB leprosy is either due to misclassification or inadequate therapy. Hence, 3-drug regimen is a simple and logical culmination of the attempt to reduce the incidence of relapse.

Evidence from a randomized controlled clinical trial of multidrug therapy paucibacillary regime (PB-MDT) plus daily clofazimine versus routine PB-MDT suggested that the proportion with persisting active skin patches was considerably lower in the clofazimine arm (7.5%) compared to the PB-MDT arm (16%), and in the six month post-PB-MDT follow up, the clofazimine group demonstrated a better response than the control group (80% versus 30%). [7] After observing better resolution of lesions, another recent study recommended a regimen that included clofazimine. [8] In a recently published 19 years retrospective analysis of 901 patients, none of the patients reported adverse effect to clofazimine.This study also highlighted the additional advantage of clofazimine in reducing the incidence of reactions and neuritis, which is a serious complication leading to deformities. [9] Unfortunately, Lockwood and colleagues have not cited these studies with more positive outcomes.

We must also add that because most of the Indian studies reported a relapse rate of more than 1 per 100 person years, the Indian leprologists have not been satisfied either with the 2 drug regimen or the duration of 6 months. A valid reason to try more drugs or a duration longer than recommended is to improve the outcome. Considering all these issues and the observed better outcome with addition of clofazimine, the Indian Council of Medical Research (ICMR) made a recommendation in 2013 for the addition of clofazimine to the WHO recommended MDT-PB schedule. [10]

A study has been cited in which 9.8% of the patients stopped therapy because of the skin pigmentation. [11] The figure though only relates to MB patients and has not been quoted correctly. Out of 293 MB patients, 7.5% of patients stopped therapy because of pigmentation. This figure cannot be logically collated for PB patients, in which the therapy is given for 6 months only. The pigmentation and xerosis (which do not occur in all cases) starts after 8 to10 weeks of therapy. And by the time the patient experiences side effects that can be considered distressing, the treatment is over. Counseling, as an essential part of ensuring regular therapy, would normally see us through.

Now, the overstated problem of stigma due to pigmentation caused by clofazimine is suggested to be due to lack of pharmacovigilance. Studying the side effects of a drug is an essential part of moral and ethical pharmacovigilance. Clofazimine has been in use for more than 60 years, and enough is known about the drug. Clofazimine has been used in higher dosage (up to 300 mg daily) for prolonged periods in type 2 reactions in leprosy, Buruli ulcer, atypical mycobacterial infections, pyoderma gangrenosum, malakoplakia, Melkersson–Rosenthal syndrome, and even nodulocystic acne [12] without serious side effects including pigmentation. Pigmentation in a few patients with fair skin could be a problem, but most Indians and patients in Myanmar/Indonesia and other African countries have a skin type III to V, in which the problem of pigmentation is hardly a concern if any. Gross skin pigmentation is also known to occur with hydroxychloroquine, hydroxyurea, minocycline, and many other chemotherapeutic agents, but it does not stigmatize the patients. So, a small dose of clofazimine for a limited period should be considered as well tolerated and safe in view of the available information. Even in the studies quoted by the authors, pigmentation due to clofazimine occurred in 10.0% to 11.1% of the treated patients.

There is also a comment about the reliability of the criteria used in studies highlighting the advantages of clofazimine to assess the reduction in the size of the lesions and reduction in the nerve thickening. Most of the leprosy clinics chart the lesions, and manual nerve palpation has been the standard part of examination over the years. Moreover, to observe the size of lesion(s) in addition to the treating physician, the patient is the best judge. Similarly, about the monitoring of adverse effects, the complaints of the patient are the best parameter and so also the regular basic laboratory investigations. Special investigations then may be asked for as and when required.

The unexplained anemia seen in patients of Goncalves and colleagues [13] cannot be attributed to clofazimine as part of treatment of PB disease. No such side effect has ever been attributed to clofazimine, neither any interaction between clofazimine and rifampicin or dapsone has been described, as the metabolic pathways of all these drugs are different. [12] If this is true, then patients on MB-MDT for 1 year or longer should have such a side effect many times more, which is not substantiated.

The statement of “do no harm” is more strongly applicable in this scenario. By introducing clofazimine, we are doing more good to the patient in bringing about early resolution of the disease and also reduce the incidence of relapse and even reactions, no harm, certainly. Moreover, in a disease like leprosy in which no clinical or bacteriological end point with a solid scientific basis is available, it is always better to do a bit “more” than to do little “less.”

The limitation of slit skin smear (SSS) in differentiating PB and MB leprosy is well known. It is a common knowledge that majority of the patients classified as MB on the basis of number of lesions are SSS negative. So, this observation does not in any way support the argument that all SSS negative patients who are actually PB are included in the MB category. All MB patients are not always smear positive. In reality, it is the other way: Not locating and counting all the lesions in intimate and/or covered areas is a fact, and, so, a MB patient is more likely to be labelled as PB. Higher incidence of relapse rates in PB disease is an indirect evidence of this misclassification.

So, in totality, benefits far outweigh the minor and mostly acceptable and/or reversible side effects of pigmentation and xerosis. More importantly these side effects do not occur uniformly in all patients. Many studies have reported dapsone to be the most common drug in causing adverse drug reactions (ADRs) in MDT (in up to 60.7% of the ADRs). [9] But nobody has ever suggested its discontinuation, because dapsone is an extremely useful drug, even though the authors do suggest the need for a safer alternative in situations of dapsone intolerance.

Moreover, in a chronic disease like leprosy, it is imperative to draw a line between trivial and transient adverse reactions and the observed advantages of a drug in this case (clofazimine).

We certainly do not endorse the call given by the authors to the National Leprosy Control Program to be careful in accepting the new WHO guidelines. This certainly will not be in the best interest of the cause of leprosy elimination.

Comments about how many extra children would have consumed clofazimine over the next many years and presenting the cumulative figure is not fair. For 16,979 children reported in the year 2017 (even if we accept that about half of them would have PB leprosy), the figure would be only about 8,500 children globally, which is not very frightening. In light of the observational studies, which have reported better lesion regression and reduced incidence of relapse with addition of clofazimine (which may even be able to prevent drug resistance), we expect all leprologists to fully support the recommendation of 3 drug regimen for use in PB leprosy. For further reducing the relapses, we draw a positive conclusion from studies using uniform MDT (presently not recommended by WHO for MB leprosy), which reportedly produced very good results in clinical regression of lesions and acceptable relapse rates even in SSS positive MB cases. [14] So, the 3 drugs now recommended for PB leprosy are bound to produce still better results in terms of patient satisfaction and reduced rates of relapse.
